# A method for detecting characteristic patterns in social interactions with an application to handover interactions

**DOI:** 10.1098/rsos.160694

**Published:** 2017-01-18

**Authors:** Nikolai W. F. Bode, Andrew Sutton, Lindsey Lacey, John G. Fennell, Ute Leonards

**Affiliations:** 1 Department of Engineering Mathematics, University of Bristol, Bristol BS8 1UB, UK; 2 School of Experimental Psychology, University of Bristol, Bristol BS8 1TU, UK

**Keywords:** social interactions, interaction patterns, social behaviour, research methods, subsequence mining

## Abstract

Social interactions are a defining behavioural trait of social animals. Discovering characteristic patterns in the display of such behaviour is one of the fundamental endeavours in behavioural biology and psychology, as this promises to facilitate the general understanding, classification, prediction and even automation of social interactions. We present a novel approach to study characteristic patterns, including both sequential and synchronous actions in social interactions. The key concept in our analysis is to represent social interactions as sequences of behavioural states and to focus on changes in behavioural states shown by individuals rather than on the duration for which they are displayed. We extend techniques from data mining and bioinformatics to detect frequent patterns in these sequences and to assess how these patterns vary across individuals or changes in interaction tasks. To illustrate our approach and to demonstrate its potential, we apply it to novel data on a simple physical interaction, where one person hands a cup to another person. Our findings advance the understanding of handover interactions, a benchmark scenario for social interactions. More generally, we suggest that our approach permits a general perspective for studying social interactions.

## 1. Introduction

Imagine handing someone a cup of coffee. Most probably you have performed this task hundreds of times. However, have you always conducted this interaction in exactly the same way? For example, have you always looked at the cup precisely one second before you let go of it? It is likely that the exact details of your actions will differ from one time to the next, but some basic patterns of behaviour are specific to the task and thus should occur again and again. For example, people may look at the other person at some point before handing them the cup. Irrespective of whether or not their interaction entails a physical/motor component, such as in the example above, individuals need to communicate with other people to conduct their interaction successfully and efficiently. In other words, they socially interact by sending and responding to many different cues and prompts [1]. Our daily lives are full of such social interactions [2], all of which require actions and communication signals to be predictable. This gives rise to characteristic patterns of actions and behaviours in social interactions [3,4]. Characteristic interaction patterns thus allow us to study the nature of social interactions (how they are conducted [5], how complex they are, how they depend on context [6]). Additionally, they provide insights into the origins of social interactions by facilitating investigations into the cognitive capabilities that are required to perform and interpret necessary behaviours [7], or into the point in the development or evolution of an organism at which these capabilities are available [8]. Understanding characteristic patterns in social interactions could lead to a number of applications. Differences in interaction patterns across individuals can be used for behavioural diagnostics (e.g. autism [9,10]), and roboticists aim to exploit predictable and highly regular interaction patterns to build machines that can interact seamlessly with humans [11–13].

The isolation of characteristic patterns in social interactions is, therefore, a crucial aspect of research into social behaviour. Reliably detecting such patterns is not trivial, as they may be masked by different sources of variation inherent in social interactions. First, differences in timing or responsiveness may result in differences in the way individuals conduct an interaction across separate instances (e.g. [5,14]), as already suggested in the example above. Second, pairs of individuals may develop a specific strategy for successfully conducting an interaction that differs from the approach taken by others. Such strategies could arise as a result of familiarity between interaction partners or they could develop as part of cultural or linguistic developments (e.g. [15]). Third, we may expect that, even if the fundamental interaction task remains unchanged, small changes to the task (e.g. handing over a full versus an empty cup) affect the way individuals conduct the interaction. Here, we present a way forward for detecting and characterizing social interaction patterns in the presence of the first source of variation. Importantly, we also show explicitly how the latter two sources of variation in interaction patterns, arising from different interaction partners and changes in the task, can be investigated. We note that a similarly general approach for detecting behavioural patterns, ‘T-pattern analysis’, has previously been developed (see [16] for a review). In principle, this approach could be used to address similar problems to our approach. However, there are important differences between our approach and this previous work which we discuss after presenting our approach.

Many previous studies on interaction patterns focus on particular aspects of social interactions and look for characteristic features within them. For example, the nature of sequential actions, such as gaps between turns in conversation [15] or the likelihood of following the gaze of interaction partners [17,18], is investigated. Alternatively, synchronous actions in social interactions are studied (e.g. joint attention [14]). Some research considers both sequential and synchronous actions, but only identifies patterns in a non-automated and, therefore, somewhat subjective way [13,19]. Moreover, often research focuses only on patterns in one person in the interaction. Examples include studies that investigate patterns in the gaze of a person who is handing an object to another person [13] or work on how underlying intentions affect the action kinematics of one interaction partner or how these intentions are perceived by another person [20]. Other research does not study interaction patterns explicitly, but uses them implicitly to distinguish between different types of social interactions or to predict the onset of social interactions. For example, machine learning techniques have been used to automatically predict an individual's intent to hand over an object [21] or to detect social interaction behaviours, such as attacking and mounting, from video recordings of fruit flies and mice [22,23]. Some more general frameworks do not consider fine-scaled interaction patterns [12]. Instead, an interaction is a combination of phases separated by decision points. For example handing an object to a person can be represented using the phases ‘approach’, ‘reach’ and ‘transfer’.

In contrast with this previous work, our approach takes account of all interaction partners and automatically detects interaction patterns that contain both sequential and synchronous actions within the same interaction. We extend ideas from database mining [24,25] and bioinformatics [26] to behavioural biology and experimental psychology. The type of social interaction pattern we investigate here can be explained by the example of turn-taking in conversations [5]. In typical conversations, first one person and then the other person speaks, and the transition between turns is communicated via behaviours such as looking at the conversation partner or a short pause [5,15]. A typical pattern in conversations could be as follows: initially, individual A speaks and B listens. Then A looks up and stops talking. This prompts B to also look up and to subsequently start talking while A listens. In other words, we investigate regular patterns in the temporal sequence of actions of two interacting people. We study such patterns in general, to investigate defining features of the interaction, and we compare the occurrence of patterns across interaction contexts. The original techniques that our approach is based on have been used to study frequent patterns in many different application areas, such as the order of items bought on a website or popular patterns in visited locations inferred from mobile phone data [27]. We argue that extending this approach to study patterns in social interaction data presents a promising avenue for future research.

We illustrate our approach by applying it to a simple and ubiquitous social interaction, where one person hands an object (a cup) to another person. Studying this interaction is interesting in its own right. Its nature makes it well suited to provide insights into the mechanisms underlying visually guided grasping of objects [28] and those underpinning gaze behaviour when performing such visually guided actions [29]. Studies into ‘Theory of Mind’ (see [30] for a review) suggest that it is this gaze behaviour that most probably serves as a communication signal between interaction partners. However, the focus of our research is not the investigation of such psychological or cognitive processes within handover interactions, but the detection and characterization of general interaction patterns and how they change across contexts. Such interaction patterns are studied intensely by roboticists, as handing over and receiving objects are important benchmark skills for robots interacting with humans [11–13]. Accordingly, a number of robotics studies utilize experiments on human-to-human handovers to inform robot design [12,13,19,21,31,32]. The dependency of interaction patterns on changes in the task and their variability across individuals are two aspects that have, to the best of our knowledge, not been investigated to date. While our findings and data on handover interactions, therefore, advance the understanding of handover interactions, we emphasize the development of our methods in this contribution.

In summary, we extend techniques from database mining to social interaction research to detect and characterize general interaction patterns in experimental data. Importantly and in contrast to previous work, we not only investigate characteristic interaction patterns *per se*, but we explicitly show how to investigate changes in interaction patterns across interaction pairs and across contexts (e.g. changing task difficulty). The remainder of this paper is structured as follows. First, we discuss what kind of data on social interactions are suitable for our approach and we introduce our novel data on physical handover interactions that we use throughout to illustrate our methods. Second, we explain our analysis for detecting characteristic patterns in social interaction data. Third, we present methods for using the detected patterns in further analysis. We show how detected patterns can be used to compare interactions across experimental treatments or across individuals. Finally, we critically discuss our approach highlighting its potential and shortcomings.

## 2. Data considerations and illustration data

### 2.1. Data requirements

Data on social interactions are collected in many different ways. Some researchers collect data in an automated way, such as using image analysis to obtain velocity profiles of the hands of interaction partners (e.g. [31,32]). Other researchers rely on manually coding the behaviour of interaction partners, such as determining their gestures or the location of their gaze relative to predefined regions of interest [13,15,19]. Our methods are designed to be general, so that they can be applied to data on social interactions that are collected in different ways. However, there are some minimal requirements on data to ensure our approach can be applied. First, it has to be possible to label the behaviour or actions of individuals in the interaction according to a number of discrete categories. This can be done qualitatively (e.g. ‘person looks at object’ or ‘person speaks’) or quantitatively (e.g. ‘person moves above speed threshold’). Second, the temporal ordering for these categorized behaviours has to be known: they can occur consecutively or synchronously, or they can overlap. Third, it has to be possible to relate the temporal ordering of behaviours from each interaction partner to the temporal orderings of all other interaction partners. In other words, the data recordings for all interacting individuals have to be synchronized. If these requirements are satisfied by data, our analysis can be applied.

### 2.2. Illustration dataset

**Figure 1.**
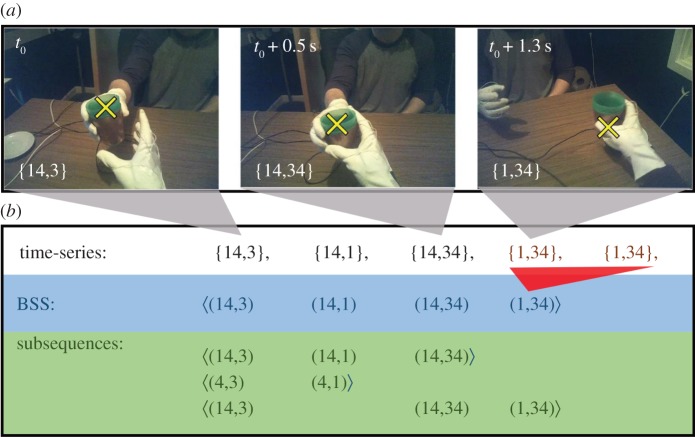
Characteristic interaction sequences (CISs) in a handover task. (*a*) Three still images from a mobile eye-tracker worn by one of the participants in our experiment. The yellow ‘X’ indicates the participant's gaze fixation point. Time is indicated in the top left corner of the images. In this interaction, the participant opposite holds the cup first, then both participants hold the cup and finally, only the participant whose perspective is shown holds the cup and completes the handover by placing the cup on a saucer at the other end of the table. We represent this interaction, using four behavioural states (see text; 1, look elsewhere; 2, look at other person; 3, look at cup; 4, hold cup). The behavioural states are shown in the bottom left corner of the still images. We always write the behavioural states for the participant who initiates the handover to the left of the comma that separates individuals' behavioural states, the behavioural states of the other person to the right. Here, the initiator does not look at the cup or the other participant for any of the still images shown. (*b*) We obtain time series of behavioural states (not shown, at a recording frequency of 24 Hz). From these, we construct behavioural state sequences (BSSs) by removing identical consecutive combinations of behavioural states (in red on first line of (*b*); ‘〈’ and ‘〉’ indicate start and end of a BSS, respectively). A BSS is a subsequence of another BSS if all sequence elements of the former are contained in the latter in the same order. We show examples for subsequences in panel (*b*). A gap of at most 1 combination of behavioural states is allowed in subsequences (bottom line in *b*). We identify a reference set of CISs in this set of BSSs by searching for subsequences that occur frequently in the set of BSSs.

To illustrate the working and potential of our approach, we record data on interactions where one person hands an object, a cup, to another person. This represents a ubiquitous social interaction that requires a high degree of temporal and spatial coordination between interaction partners [12,13]. We record complete handover interactions experimentally with 11 pairs of participants from 0.5 s before the cup is first picked up until 0.5 s after it is put down again (see electronic supplementary material, table S1 for details on participants). To illustrate how small changes in the nature of the task could affect interaction patterns, we record 20 replicate handover interactions for each participant pair separately with an empty cup and with a cup filled to the brim with dry rice grains (two experimental treatments). We, therefore, obtain data for a total of 440 handover interactions (220 each for treatment levels full and empty). In the experiment, handover interactions are initiated by a visual–auditory signal that ensures short 1 s intervals between consecutive handovers. Throughout the experiment, both participants wear a mobile eye-tracker. We manually annotate videos recorded from eye-trackers at 24 Hz, determining for each time point (frame) whether participants hold the cup, as well as whether participants are looking at the cup, the face of the person opposite to them or neither of these regions of interest (figure 1*a*). We use the time points when both participants hold the cup to synchronize data from the two participants in each pair. Data collection using our mobile eye-trackers was prone to data loss and the data introduced above are, therefore, only a selection of all data collected. Full details on this and all experimental procedures can be found in the supplementary information.

As we use these data primarily to illustrate our approach, we defer a discussion on our choices in data collection with regard to capturing salient aspects of handover interactions to the end of this contribution. At this point, we justify our experimental set-up by explaining how it allows us to illustrate key features and capabilities of our methods.

We expect that visual attention on the object that is being handed over and on the interaction partner are likely to be important aspects of characteristic patterns in handover interactions. Attending to objects is important for the visually guided grasping of objects [28], which is an integral part of handover interactions. We also expect that individuals attend to the other person to check that they are engaged with and attending to the task at hand [29]. By recording if the momentary visual fixation of individuals is directed at the object, the interaction partner or elsewhere, we can determine how frequently and in what order these behaviours occur in handover interactions.

As we adjust the difficulty of the handover task by varying how full the cup is, we can test if interaction patterns are affected by these changes in the task. Previous work predicts that more challenging tasks require more action planning to conduct a successful interaction [33,34]. This might only affect the time taken to complete the interaction and lead to extended time periods covered by the same interaction patterns (patterns get stretched in time); alternatively, this could result in new interaction patterns that could indicate a change of strategy for conducting the interaction.

Finally, as we have conducted the experiment with different pairs of participants, we can explore if there are differences in characteristic interaction patterns between interaction pairs. Our participant pool is fairly homogeneous (mostly undergraduate students), and the interaction we study is ubiquitous and simple. Therefore, in the absence of an established literature on this topic, we do not expect large discrepancies in interaction patterns across participant pairs.

## 3. Methods for detecting patterns

In this section, we describe our approach for detecting characteristic patterns in social interaction data. For each step in our analysis, we describe the results of applying it to our illustrative handover data. We first describe our approach for finding patterns that occur frequently and subsequently, we present ways in which these patterns can be investigated further.

### 3.1. Behavioural state sequences

The first key concept in our analysis is to represent a social interaction as a sequence of, potentially co-occurring, behavioural states. It is important that we can always associate at least one behavioural state with each of the interacting individuals. To guarantee this, it is always possible to define one behavioural state to occur precisely when one or more other behavioural states do not occur. We illustrate this for our handover data, where we define four behavioural states, labelled numerically:

1. participant looks elsewhere;
2. participant looks at the face of the other participant;
3. participant looks at the cup; and
4. participant grasps the cup.

At each observation time point, we can therefore describe our data in terms of these behavioural states (figure 1*a*). For example, if in a frame one participant looks elsewhere (state 1) and grasps the cup (state 4) while the other looks at the cup (state 3), we can write {14,3}, where the behavioural states for the two participants are separated by a comma and ‘14’ indicates that both states 1 and 4 apply to one participant. To ensure that this notation is consistent across experimental sessions, we always write the behavioural states of the participant who initiates the handover on the left-hand side of the comma. From our choice of behavioural states, it is clear that we can identify at least one behavioural state for each individual at all times (states 1–3 are mutually exclusive, but one of them always applies).

This data labelling procedure transforms social interaction data into a time series of symbols (behavioural states) for each interaction partner. As individuals display behaviours for a period of time, it is likely that these time series contain many identical consecutive entries (see figure 1*b* for an example). The number of identical consecutive entries captures the duration a particular combination of behavioural states is displayed. The second key concept in our analysis is that we do not consider these durations. We do not wish to suggest that the duration for which behaviours are displayed is not important. Instead, we emphasize that, in our analysis, we focus on detecting interaction patterns independently of variation in such durations and focus on changes in behaviour. Therefore, we construct behavioural state sequences (BSSs) from each time series representing an interaction (figure 1*b*). In these sequences, we capture changes in behavioural states instead of the duration participants adhere to different behavioural states. We require that consecutive elements in a BSS must differ by at least one behavioural state for at least one participant. For example, the four entries {1,2}, {14,2}, {14,2} and {2,14} in a time series give rise to the BSS ‘〈(1,2)(14,2)(2,14)〉’. We denote the start and end of a BSS with the symbols ‘〈’ and ‘〉’, respectively. Sequence elements are given in round brackets. The length of a BSS is given by the number of its sequence elements. In this way we construct 440 BSSs from our handover data, one for each handover interaction included in our analysis. The average length of the BSSs in our data is 27.6 sequence elements (maximum and minimum lengths were 69 and 7, respectively).

### 3.2. Finding frequent sequences in behavioural state sequences

The next step in our analysis is to find patterns of behavioural states that occur frequently in the BSSs. To achieve this, we need to define what we mean by a particular pattern of behavioural states ‘occurring in’ or ‘being contained in’ a BSS.

We begin this definition by considering the shortest possible pattern: let *a* be a sequence element of a BSS (i.e. a sequence of length 1). We define that the sequence element *b* is contained in *a* if *a* includes at least the same behavioural states for each individual as *b*. For example, (1,2) is contained in (14,24) or (1,2), but not in (2,1), following the notation established above.

Based on this definition, we can now consider longer patterns: consider the sequences *A* *=* 〈*a*_1_*a*_2 _… *a_k_*〉 and *B* *=* 〈*b*_1_*b*_2 _… *b_l_*〉, where *b*_1_, *b*_2_, etc. are sequence elements and the indices indicate the order of sequence elements. We say that *B* is a subsequence of *A* if we can find integers *i*1 < *i*2< ⋯ < *il* such that the sequence element *b*_1_ is contained in sequence element *a_i_*_1_, *b*_2_ is contained in *a_i_*_2_, etc. *B* is a subsequence of *A* with a gap of at most *δ* (*δ* *≥* 0) if we additionally have *io − ip* *≤* *δ* *+* 1 for all *p* *=* 1*, … ,l − *1 and *o* *=* *p* *+* 1. In other words, we define that a BSS *B* is a subsequence of *A* if all sequence elements of *B* are contained in *A* in the same order as they appear in *B* with a gap of at most *δ* sequence elements between consecutive sequence elements (figure 1*b*). For example, if *δ* *=* 1, then 〈(1,2)(2,1)〉 is a subsequence of itself, or of 〈(1,2)(3,3)(2,14)〉; but it is not a subsequence of 〈(1,2)(3,3)(3,1)(2,14)〉 (however, for *δ* *=* 2*,* it would be a subsequence).

The gap length *δ* is a parameter in our analysis. Low values of *δ* imply that subsequences must be contained in other sequences with few or no other sequence elements between elements of the subsequence. The gap length can, therefore, be interpreted as controlling the amount of noise or spurious sequence elements in BSSs we allow for in our analysis. It can be set very conservatively (e.g. *δ* = 0), to ensure subsequences occur in exactly the same way in all observations investigated. Alternatively, for more exploratory analysis, high values of *δ* imply that we allow a considerable number of spurious sequence elements between elements of subsequences. As we investigate a highly repetitive and simple task in our illustrative data, we expect few spurious sequence elements in interactions and we, therefore, set *δ* = 1 throughout.

We now demonstrate how to use this concept of subsequences to find characteristic patterns in our BSSs. We build on previous work on mining for sequential patterns in datasets of sequences [24,25] by using a modification of the ‘Apriori algorithm’ [24] to find all frequent subsequences (FSs), i.e. sequences that are a subsequence of at least a minimum fraction *θ* of all BSSs in the dataset. As explained above, we investigate interaction patterns that include changes in behavioural states. Therefore, we require that consecutive sequence elements in FSs are not identical. Our algorithm is general and can be applied to datasets with different numbers of behavioural states. It consists of the following four steps:

(i) We find sequence elements that occur in a fraction *θ* of all BSSs (i.e. FSs of length 1). All FSs of length longer than 1 must be composed of these sequence elements. In this step, all sequence elements are considered, even if some are contained in others. For example, if (12,1) occurs in a fraction *θ* of all sequences, then so do the sequence elements (1,1) and (2,1).
(ii) Suppose that step (i) is completed and that we have found all FSs of length *n* (*n* ≥ 1). Call these *A_i_^*n*^*, where the index *i* runs over all FSs of length *n*. Create candidate subsequences of length *n* + 1 by adding all allowed sequence elements from step (i) to the end of all *A_i_^*n*^*. We do not allow identical consecutive elements in the generation of candidate subsequences.
(iii) For each candidate subsequence of length *n* + 1, we compute the fraction of BSSs in the dataset of which it is a subsequence. If this fraction is greater than or equal to *θ*, include this candidate subsequence in the set of FSs of length *n* + 1, *A_i_^*n*^*^+1^.
(iv) We repeat steps (ii) and (iii) until we reach a value of *n* for which we find no more FSs. The upper limit of *n*, regardless of the value of *δ* or *θ* is given by the length of the longest BSS in the dataset.

An application of this algorithm to a small illustrative dataset is presented in table 1 and an implementation of this algorithm is available alongside our data (see Data accessibility). As this algorithm is a modification of earlier work [24], it is known that this procedure finds all FSs for a given dataset of BSSs.

**Table 1.** Output of algorithm for finding frequent subsequences (FSs) when applied to an illustrative dataset. The first column shows a set of three behavioural state sequences (BSSs). We set the threshold for detecting FSs to *θ* = 1 (i.e. FSs have to occur in all BSSs) and the allowed gap in this procedure to *δ* = 0 (see main text for description of parameters). From left to right, we show candidates for FSs of increasing length. The set of FSs of length *n* is denoted by *A^n^* (see also main text). FSs, i.e. candidates that occur in the number of BSSs as required by *θ*, are underlined. Candidates of length *n *> 1 are constructed by adding FSs of length 1 to candidates of length *n *−* *1, under the condition that consecutive sequence elements are not identical. For example, the two FSs of length 1 give rise to two candidates for FSs of length 2, one of which, 〈(1,1)(2,2)〉, is an FS.

set of BSSs	candidates of length 1 (*A*^1^)	candidates of length 2 (*A*^2^)	candidates of length 3 (*A*^3^)
〈(1,1)(2,2)〉	〈(1,1)〉	〈(1,1)(2,2)〉	〈(1,1)(2,2)(1,1)〉
〈(1,1)(2,2)(1,2)〉	〈(2,2)〉	〈(2,2)(1,1)〉	
〈(1,12)(2,2)〉	〈(1,2)〉		
	〈(1,12)〉		

The threshold *θ* is another parameter in our analysis. By choosing different values for *θ*, we can decide in how many of our observed interactions a subsequence has to occur to be considered an FS. There are no general rules for setting this parameter, as this depends on the scientific question. Our application of the analysis to our handover data suggests one way of approaching this problem.

When applying this analysis to our handover data, we need to decide if we investigate data from the two experimental treatments (full versus empty cup) separately, or not. Here we decide to conduct our FS analysis on the combined dataset of all 440 BSSs. We also have to decide on the threshold level *θ* we use. On the one hand, we want to investigate patterns that are characteristic of a handover interaction. This requires high levels of *θ*. On the other hand, we want to study differences between two experimental treatment levels, and only for low values of *θ* we can capture all patterns that may differ between treatment levels. Here, we present results for *θ* = 0.4, but it is possible to show that specific results are robust over a range of values for *θ* (see below).

The result of applying our analysis to the set of 440 BSSs from our handover interaction data is a set of 4437 FSs of different lengths (24, 237, 858, 1429, 1155, 635 and 99 subsequences of lengths 1, 2, 3, 4, 5, 6 and 7, respectively). In principle, it is possible to use this set of sequences for further analysis, and all of the analysis we describe in the remainder of this contribution could be applied to this set of FSs. However, we will argue below that many of these sequences do not add substantial information about characteristic patterns in the observed interactions and we, therefore, focus our further analysis on a selection of these FSs.

### 3.3. Characteristic interaction sequences

The analysis we have described so far produces potentially large sets of FSs. From the way in which we search for these sequences, it is clear that some of them will be of length 1 and that some of them may be subsequences of other FSs. FSs of length 1 tell us what the interaction partners are doing at the same time. While this is interesting and allows us to investigate the nature of synchronous actions for participants, it fails to capture sequential actions in interactions. Furthermore, longer FSs are composed of FSs of length 1 and, therefore, also contain information on synchronous actions. For these reasons, it may not be necessary to consider FSs of length 1 in further analysis. Some FSs may be subsequences of other FSs. The information about characteristic patterns provided by such subsequences is already contained in other FSs, and therefore, it could be argued that only FSs that are not a subsequence of any other FS should be considered in further analysis.

This approach of only selecting FSs that add substantial information about characteristic patterns for further analysis has the advantage that the overall number of FSs considered in the analysis is likely to be greatly reduced. For illustration, we use this approach on our handover data. We call sequences in this reference set characteristic interaction sequences (CISs). Table 2 presents a summary of all acronyms and parameters used in our analysis.

**Table 2.** Summary and description of acronyms and parameters used in analysis. See also figure 1 and table 1 for the relations between BSS, FS and CIS. See main text for detailed descriptions.

acronym/ parameter	brief description
BSS	behavioural state sequence: sequence that is constructed from a time series of behavioural state combinations by removing identical consecutive elements. Behavioural states are a discrete qualitative or quantitative coding of behaviour in a social interaction (e.g. ‘look at object’ or ‘hold object’)
*δ*	allowed gap length in sequence elements used when determining if one BSS is contained in (is a subsequence of) another BSS. We use *δ* = 1
*θ*	threshold used when determining if sequences appear frequently in a given dataset of BSSs. We use *θ* = 0.4
FS	frequent subsequence: sequential pattern of behavioural state combinations without identical consecutive sequence elements that is a subsequence of a fraction of at least *θ* of all BSSs in a given dataset
CIS	characteristic interaction sequence: for a given dataset, this is the set of FSs of length > 1 that are not a subsequence of any other FS found in the data

From our handover data, we find a reference set of 852 CISs with an average length of 4.6 sequence elements (a file containing all CISs is available alongside our data; see Data accessibility). These CISs can cover a complete handover (i.e. first one, then both, then the other participant holds the cup) or only part of the interaction (e.g. only one participant holds the cup, but visual attention of both participants changes). In the following, we use this reference set of CISs in our methods description and further analysis of our handover data. However, the methods are general and also work on the larger set containing all FSs.

### 3.4. Summarizing features of characteristic interaction sequences

Once we have found a set of CISs (but using FSs instead is also possible, as described above), we would like to further investigate the patterns we have detected. Here, we present techniques of pursuing two main lines of investigation. First, we assess if the patterns we have found occur in the data more often than we would expect by chance. Second, we investigate features of the detected patterns in terms of the behavioural states they contain.

Often it is desirable to test whether CISs are a by-product of a high degree of redundancy or repetition in combinations of behavioural states. This can be achieved by randomizing the observed data. Different randomizations are possible and each of them represents an assumption for how interaction data, represented as BSSs, arise by chance. One approach that we use here is to test the null hypothesis that the number of times a CIS occurs in the observed BSS is no higher than we would expect if behavioural states in BSS are reordered at random. In this permutation test, we reorder the behavioural states in a BSS separately for each individual. This affects synchronous as well as sequential patterns in BSSs. We compute *p*-values by counting how many times across 10 000 permutations of our BSSs a CIS occurs more frequently than in the original data (we include the original data in this analysis; so the minimum *p*-values are 1 × 10^−4^). As with all permutation tests, the number of permutations performed should be large. But often it is impossible to evaluate all possible permutations of the data.

We apply this analysis to the 440 BSS and the 852 CIS we obtained from our handover experiment. We find that all but 28 of the CISs occur in the data more frequently than we would expect under the null hypothesis described above (electronic supplementary material, figure S1*a*). This shows that the CISs we detected are not simply a result of repetitions or redundancy in behavioural states. In the following, we continue to consider all CISs in our analysis, because the number of CISs with non-significant *p*-values is small and because these CISs may still capture behaviourally meaningful aspects of the interaction or reveal differences in behaviour across experimental conditions.

To investigate the features of CISs in terms of the behavioural states they contain in more detail, we record general properties of CISs that can be interpreted easily and are behaviourally meaningful. First, we can record which of the four behavioural states are contained within a CIS. Second, we can record if a CIS contains sequence elements representing synchronous actions, such as (1,1) or (4,4). Third, we can record the length of CISs. This provides a measure for the number of sequence elements, and therefore, for how many separate steps the interaction pattern involves. Fourth, we can record if the behavioural state patterns for all interaction partners change in a CIS. This can be used as an indication for patterns in which all interaction partners are actively involved rather than passively observing the actions of others. Fifth, we can record if, in a CIS, first exclusively one and then exclusively the other interaction partner displays a particular behavioural state. For example, in our handover data, we can investigate if a CIS contains a change in grasp of the cup (state 4) or in visual attention on the cup (state 3). We define the former as a CIS in which initially only one participant holds the cup and subsequently only the other participant holds the cup (the synchronous action (4,4) can be, but does not have to be contained in the CIS). A shift in visual attention is defined similarly. Exploring this kind of feature in CIS could be useful when investigating causal relations between specific interaction states of the interacting partners. Depending on the interaction investigated, other features may be of interest (e.g. particular sequences of behavioural states). For the purpose of illustration, we focus on the above-mentioned properties.

Computing these properties for our handover data reveals, interestingly, that none of the CISs contains behavioural state 2 (looking at the other person; see electronic supplementary material, table S2 for a summary of all properties). All other behavioural states are represented in over 50% of CISs (over 98% for states 1 and 4). This suggests that looking at the interaction partner is not a characteristic component of handover interactions which confirms previous findings [12,13]. CISs were on average 4.6 sequence elements long (maximum length: 7) and were, therefore, much shorter than the BSS they were detected in. The synchronous presence of behavioural states in the two interaction partners is frequent in CISs for state 1 (both partners looking elsewhere, in approx. 87% of CISs), relatively frequent for state 4 (joint holding of cup, approx. 40%) and rare for state 3 (both looking at the cup, approx. 1%). More than three-quarters of all CISs (approx. 75%), included changes in behavioural states for both interaction partners. This suggests that the patterns are characteristic of handover interactions, rather than simply capturing the actions involved in picking up and placing down a cup. However, to comprehensively assert this suggestion, it would be necessary to compare the patterns we find to patterns in a control condition in which one participant picks up a cup and puts it back down again without interacting. Fewer than half of all CISs (approx. 41%) include a change in grasp. Therefore, most of the patterns we find do not simply reflect the physical handover in which first one and then the other participant holds the cup. Only about 4% of CISs include a change in visual attention on the cup, such as 〈(1,3)(3,1)〉. These findings show that even a simple, highly regular social interaction, such as passing a cup from one person to another, contains a large number of distinct patterns that occur frequently in interactions.

## 4. Methods comparing detected patterns across treatments and participants

So far, we have introduced methods for detecting patterns that occur frequently in interaction data and we have suggested approaches for further investigating properties of the detected patterns. This analysis is interesting in its own right, but as discussed in the introduction, it is important to establish if and how interaction patterns differ across experimental treatments or participants. Extending the scope of our pattern detection analysis in this direction is the subject of this section. The key concept in our approach is to assess how often patterns occur in different treatments or participants. As before, we illustrate the methodology by applying it to our data on handover interactions.

### 4.1. Differences in interaction patterns across experimental treatments

Our interaction pattern detection results in a set of CISs (recall that this set could be composed of all FSs instead of a selection, as discussed above). By definition, these CISs occur in many, but not necessarily in all of the BSSs we record. To investigate differences in interactions across treatments and across interaction pairs, we assess whether or not CISs occur in (are subsequences of) BSSs. The precise details of this approach depend on the context (e.g. number of treatment levels investigated) and are best illustrated using an example. Therefore, we describe the approach for investigating our handover data. This approach can easily be extended to different data and contexts.

**Figure 2.**
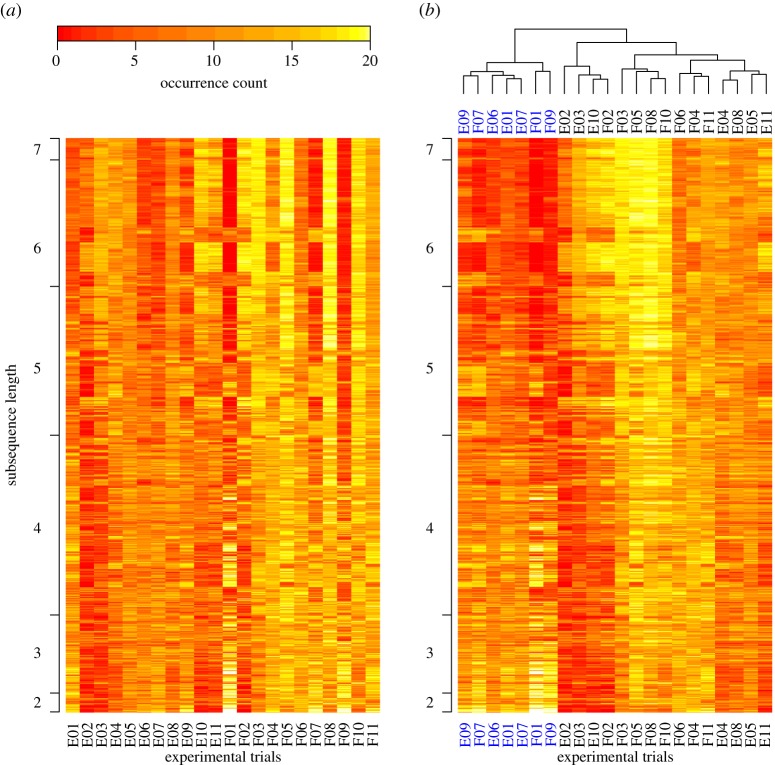
Prevalence of characteristic interaction sequences (CISs) across contexts. (*a*) Occurrence counts for all 852 CISs. Each row shows the counts for one CIS, and we additionally indicate the length of CISs along the *y*-axis. We show in how many of the 20 handover interactions recorded for each experimental run the CIS occurs. Experimental runs E01–E11 are for an empty cup and runs F01–F11 for a full cup (participant pairs in trials E01 and F01 are the same). (*b*) Same data as in (*a*), but arranged according to a hierarchical clustering of occurrence count similarities. Vertical distances in the dendrogram indicate relative between-cluster distances. We denote experimental runs in one cluster, *α*, in blue.

We aggregate occurrence data (i.e. whether or not a CIS is in BSSs) over replicate observations of interactions, but separate by treatments and participant pairs. Recall that we included 11 participant pairs in our analysis. Each pair conducted handover interactions in two treatments (full versus empty cup) and repeated these interactions 20 times. We call the 20 replicate interactions of a participant pair in one treatment an *experimental run*. Aggregating these data as described yields 22 occurrence counts for each CIS (one per experimental run). We combine these counts in 22 vectors with one entry for each of the *m* = 852 CISs. The entries of the vectors take values between 0 (if the corresponding CIS occurs in none of the handover interactions of the corresponding participant pair in the corresponding treatment), and 20 (if it occurs in all handover interactions). We call these vectors *m-*vectors. Figure 2*a* shows *m*-vectors for each experimental run.

As a measure of similarity between the CIS occurrence counts for two experimental runs, we use the Euclidean distance between the two corresponding *m*-vectors. Low values for distances imply that the number and type of CISs found in two experimental runs are similar. We can use this concept of similarity in occurrence counts to address a number of questions. First, we will use this concept to show that our analysis produces similar results across a range of thresholds, *θ*, for detecting patterns. Second, we will use occurrence counts to show that there are global differences in the occurrence of patterns across experimental treatments. Third, we will assess if there are individual patterns that occur significantly more frequently in either of the treatments.

To show that our analysis produces similar results across a range of thresholds for determining FSs, *θ*, we report the Spearman rank correlation between pairwise distance matrices computed from *m*-vectors over a range of *θ*. For each value of *θ*, we obtain a 22 × 22 matrix of pairwise distances between all 22 *m*-vectors. When computing the correlation between these matrices for different values of *θ*, we only use values above the leading diagonal. Specifically, we are interested in values of *θ* in the interval [0.25,0.5]. We choose the upper bound of this interval, because any CIS that occurs in more than 50% of our data have to occur for both treatment levels. This analysis would thus exclude patterns that only occur for one treatment level. The lower bound of this interval ensures that the patterns we consider are still reasonably representative of a handover interaction between two individuals. We find that within this interval of values for *θ*, CIS occurrence similarity is conserved (as indicated by high correlation coefficients; see electronic supplementary material, figure S1*b*).

We now turn to investigate differences in interaction patterns across experimental treatments. The average duration of handover interactions with full cups is significantly longer than with empty cups (mean ± s.d.: empty 4.61 ± 0.54 s; full 6.47 ± 0.87 s; Wilcoxon signed-rank test, *W* = 66, *p* = 0.001, comparing averages across 22 experimental runs). This leads to the question of whether participants show the same interaction patterns in both treatments, perhaps performed more slowly when the cup is full, or if there is a change in the interaction patterns displayed across treatments.

We extend the concept of measuring the similarity in CIS occurrence counts to compare the prevalence of interaction patterns across experimental treatments. To test if the difference between the two treatment levels is higher than we would expect by chance, we conduct a permutation test on our data. Our test statistic is the sum of all 121 pairwise distances between *m*-vectors in the two treatment levels (from 11 participant pairs for each treatment). We use the sum of all distances to ensure that we measure similarity within and between participant pairs. We randomize our data by switching the treatment level label for *m*-vectors within experimental pairs. In other words, for each participant pair we can either swap the data from the full cup treatment with the data from the empty cup treatment, or not. This yields 2^11^ possible assignments of treatment labels to experimental runs. We evaluate our test statistic for all possible treatment-level permutations and report the fraction of values that are higher than or equal to the observed value as our *p*-value. This analysis yields that occurrence counts differ significantly across treatments and are generally higher for the full cup treatment (*p* = 5.86 × 10^−3^).

Having established that there is a global difference in CIS occurrence counts across treatments, we now identify individual CISs that occur in significantly different numbers across treatment levels. We construct contingency tables for the occurrence of each CIS. In these tables, we record in how many (out of all 440) BSSs the CIS occurs, split up according to treatment levels. We then apply *χ*^2^-tests on the contingency tables with 1 d.f. for comparisons over treatment levels (see a discussion on this analysis below). This approach is inspired by techniques used in bioinformatics [26]. We use a threshold of 0.001*/m* (Bonferroni's correction for multiple comparisons, where *m* *=* 852 is the number of CISs in the reference set) for *p*-values in these tests to identify CISs that occur in significantly different numbers across treatment levels (a threshold of 0.01/*m* produces the same results, qualitatively). For each of these CISs, we determine for which treatment level the occurrence frequency is the highest. In this way, for each treatment level we obtain a set of CISs that are more likely to occur for this treatment level, compared with the other treatment levels. We then compare properties of these sets of CISs against the remainder of CISs.

It is unlikely that BSSs within experimental runs are fully independent. For example, participant pairs may interact in a consistent way. Therefore, it could be argued that not all assumptions for applying *χ*^2^-tests to our data as described above are satisfied. However, we only use our approach to select sets of CISs instead of individual FSs. We suggest this approach is more intuitive and captures the differences in the occurrence of CISs better than other approaches, such as thresholds for differences in occurrence.

Applying this analysis to our handover data, we find that 67 CISs occur significantly more often in interactions with full cups but none occur significantly more frequently in the empty cup treatment (table 3; electronic supplementary material, figure S2). These numbers imply that many CISs are equally likely to occur in both treatments.

**Table 3.** Features of characteristic interaction sequences (CISs) that occur more frequently in particular contexts. For each CIS, we construct a contingency table for how often it occurs in the behavioural state sequences (BSSs) for different treatments (figure 2*a*) or clusters (figure 2*b*). From these tables, we determine whether the CIS occurs significantly more frequently in one treatment or cluster. We summarize how CISs that occur significantly more frequently in BSSs for one treatment or for one of the clusters differ from the remaining CISs. We show the fraction of CISs that contain a feature in the set of CISs that occur more frequently in the cluster/treatment minus the fraction of CISs that contain a feature in the remaining CISs. For example, ‘0.8–0.5’ indicates that 80% of CISs that occur significantly more frequently contain a particular feature, whereas only 50% of the remaining CISs contain this feature. We show differences in the following features: behavioural state occurrence, synchronous occurrences of behavioural states, change in grasp (i.e. first one and then the other participant holds the cup), change in visual attention on the cup (i.e. first one and then the other participant looks at the cup) and average pattern length.

	full cup treatment	cluster *α* (E09, F07, E06, E01, E07, F01, F09)
number of patterns that occur significantly more frequently	67	51
contains 1	1.000–0.997	0.961–1.000
contains 3	0.672–0.557	1.000–0.538^a^
contains 4	1.000–0.985	0.882–0.993^a^
contains (1,1)	0.493–0.902^a^	0.275–0.908^a^
contains (3,3)	0.000–0.010	0.157–0.000^a^
contains (4,4)	0.388–0.400	0.216–0.411
contains change in grasp, e.g. (1,4)(4,1)	0.015–0.441^a^	0.020–0.432^a^
contains change in visual attention on cup, e.g. (1,3)(3,1)	0.045–0.043	0.490–0.015^a^
average pattern length	3.6–4.7^a^	3.0–4.7^a^

^a^Indicates whether the absolute value of the difference in feature prevalence is higher than we would expect by chance (permutation test on CISs included in a set of CISs that occur more frequently; significance threshold 0.01 with Bonferroni's correction for multiple (nine) comparisons; 100 000 permutations)

Next, we compare the prevalence of features in CISs that occur more frequently in handovers with full cups to their prevalence in the remaining CISs (table 3). We find that fewer of the former CISs contain a synchronous presence of state 1 (looking elsewhere) than the latter CISs (49% versus 90%). Further, the CISs that occur significantly more frequently in handovers with full cups are, on average, over one sequence element shorter, and very few of them cover a complete handover that includes a change in grasp (1.5% versus 44% of CISs). Other differences between the sets of CISs are no different from what we would expect by chance (table 3).

Consider the following three phases of a handover: one person holds the cup, both hold the cup, the other person holds the cup. Our findings suggest that patterns within each of these phases, but not patterns spanning across the phases, occur more frequently in handover interactions with full cups. In other words, participants appear to shift their attention more often between gazing at the cup (state 3) and looking elsewhere (state 1) during the different phases when handing over a full cup. This observation is in line with previous work suggesting that more action planning is required to successfully conduct more challenging interactions [33,34].

### 4.2. Differences in interaction patterns across participants

We can also use CIS occurrence counts to investigate how interaction patterns differ across participant pairs. Recall that, for each participant pair, we have data from two experimental runs, one per treatment, and that the occurrence of CISs in these data is captured in *m*-vectors. To investigate if the differences in handover interactions between experimental treatments capture all major differences between experimental runs, we keep data from the two experimental treatments separate. We use the pairwise distances between *m*-vectors to cluster experimental runs with an effective hierarchical clustering algorithm (‘hclust’ in the R programming environment [35], default settings). Hierarchical clustering constructs cluster by successively merging the most similar clusters until only one cluster remains, starting with all elements in separate clusters. This analysis also yields between-cluster distances. For our data, we find two large clusters of experimental runs (figure 2*b*). One of these, which we denote as cluster *α* (figure 2*b*), is fairly homogeneous in terms of within-cluster distances between experimental runs. The other larger cluster is more heterogeneous and can be separated into several clearly distinguishable smaller sub-clusters. We further investigate these clusters and focus on the more homogeneous cluster *α*. While it would be interesting to investigate the structure of the other large cluster in more detail, the amount of data available does not suffice to make any clear statistically founded statements.

Cluster α in figure 2*b* contains experimental runs from both treatments that are matched according to participant pairs (e.g. ‘E01’ and ‘F01’), with the exception of participant pair 6 in the empty treatment (‘E06’). This suggests that participant pairs included in this cluster interact differently to others, but we could not find any clear pattern in terms of gender composition, familiarity or treatment order for participant pairs that could explain this cluster (see electronic supplementary material, table S1).

To establish how the experimental runs in cluster *α* differ from the remaining ones, we next use occurrence counts to identify CISs that occur more frequently in this cluster. Similar to our analysis across treatment levels above in §4.1, we construct contingency tables for each CIS. We first identify CISs with a significant *p*-value in a *χ*^2^-test on this contingency table (as above). These CISs are either over- or under-represented in cluster *α*. We only select the former CISs that occur disproportionately often in cluster *α*.

We find 51 such CISs (table 3; electronic supplementary material, figure S2). As before, we compare the prevalence of features in CISs that occur more frequently to the prevalence of features in the remaining CISs. We find that all CISs that occur significantly more frequently in cluster *α* include visual attention on the cup (state 3), compared with about 54% of the remaining CISs. In addition, these CISs include all CISs that contain synchronous occurrences of state 3 and most of the CISs that contain a change in visual attention on the cup (table 3). This suggests that the experimental runs in cluster α represent handover interactions that are characterized by visually guided action (visual attention on the cup).

For the reasons discussed above, we do not investigate the larger cluster in detail. The results in table 3 and electronic supplementary material, figure S2, give an indication of what characterizes the CISs that occur more frequently in the experimental runs in this larger cluster. For example, these CISs are likely to be longer than average, contain more synchronous occurrences of state 4 (grasp cup) and are more likely to cover a complete handover in which first one and then the other participant holds the cup. The amount of data included in our analysis means that sub-clusters of the larger cluster are composed of small numbers of experimental runs, making it difficult to test hypotheses about this structure statistically. However, it is interesting to note that the order in which participant pairs completed our experimental treatments could affect the interaction patterns we observe. The four experimental runs in the full treatment for participant pairs who completed this treatment first all have similar CIS occurrence profiles (runs ‘F03’, ‘F05’, ‘F08’ and ‘F10’ in figure 2*b*; see also electronic supplementary material, table S1). Specifically, figure 2*b* suggests that CISs of length 5 or longer occur more frequently in these experimental runs. Our data are insufficient to establish such an effect conclusively, but it would be interesting to investigate this further in future work.

## 5. Discussion

In summary, we have presented a general method for detecting characteristic patterns in social interactions. Moreover, we have suggested approaches for further investigating the detected patterns, as well as using the detected patterns to compare interaction patterns across contexts, such as experimental treatments or interacting individuals. Throughout, we have demonstrated the usefulness and potential of our methods by applying them to a novel illustrative dataset on physical handover interactions.

A similarly general approach for detecting patterns in behavioural data, T-pattern analysis, has previously been suggested (see [16] for a review). We do not wish to suggest that our approach is superior. Rather, we think that the different techniques are complementary, as they identify patterns in different ways. The crucial data for T-pattern analysis are time intervals between point observations (e.g. the time that passes between the start and endpoints of behaviours). Statistically significant temporal patterns of behaviours are detected by assessing if behaviours appear more often within critical time intervals from each other than expected by chance. Such an analysis is particularly suited to find repeated patterns within long sequences of behaviours rather than to find characteristic patterns in many instances of short sequences as in our approach. Moreover, T-pattern analysis only detects patterns that occur more frequently than expected by chance. This detection thus relies on hypotheses of what represents a suitable randomization of data, similar to the tests we performed in §3.4. Furthermore, from our methods description above, it is clear that we do not use temporal information in the same way to detect interaction patterns. We illustrate conceptual differences between the two approaches using the following example: suppose that we observe exactly the same sequence of actions in two different pairs of interacting individuals. However, one pair takes twice as long to complete each action than the other pair. Our analysis does not take the duration for which behaviours are displayed into consideration directly and would return the sequence of actions observed as a pattern that occurs frequently in the collection of observed interaction sequences. T-pattern analysis would be applied separately to each observation, and it would find the same pattern in both observations, but for different minimal values of the critical time-interval parameter. Thus, considerations in setting values for analysis parameters are dissimilar in the two approaches. We suggest that while the two approaches can in principle be used to investigate similar problems, they differ substantially and are, therefore, complementary.

Our approach relies on representing salient features of the handover interaction by recording the presence or absence of four behavioural states. Many additional behavioural states, such as ‘participant extends arm’, could be defined and measured to represent the interaction in more detail (e.g. [12,13] for additional behavioural states). This leads to the question of how many behavioural states should be included in an analysis. Our findings would not be invalidated by recording additional behavioural states: the patterns we have found would still be subsequences of sequences with additional behavioural states. In addition, our approach presents a way of testing the relevance of particular behavioural states for patterns in interactions: looking at the other person (state 2) turned out not to be a characteristic behavioural state in our handover interaction data. However, it is likely that additional behavioural states would allow further insights into the mechanisms underlying handover tasks and inter-individual differences in executing such tasks. Ultimately, the question of how many states to measure depends on the question investigated. Only when interaction patterns are used to predict particular actions with a certain degree of accuracy, it may be possible to give a definitive answer on the required number of behavioural states that need to be recorded [21]. To avoid making arbitrary and uninformed decisions on which behavioural states to include in an analysis, we suggest that this process should be conducted in an objective and ideally automated way (e.g. by using statistical methods to categorize measured behaviour, as discussed below). At this point, it should be noted that recording additional behavioural states increases the computational demand of our algorithms. We have not made any attempts to increase the computational efficiency of our algorithms, but previous work on FS mining provides many approaches for how to do so [36].

It is important to reiterate the distinction between our analysis and approaches that investigate the duration for which people show certain behaviours or the time intervals between different actions [14,15]. We focus on the sequence of changes in the behavioural states of individuals, regardless of the duration for which they are adhered to. However, we do not wish to imply that durations are not important in social interactions. While our approach does not take account of durations, approaches using averaged durations may average out important behaviours that only occur for short times.

We illustrate our approach by applying it to novel data on physical handover interactions. We suggest that our results advance our understanding of patterns in this interaction. We detect many distinct, yet frequently occurring patterns. While many of them are preserved across contexts, some depend on the experimental treatment (cup fullness) or on the individuals involved (cluster *α*). In particular, we find that patterns which occur more frequently in handover interactions with full cups typically do not cover the entire interaction, but only part of it (e.g. only one person touches the cup, or both participants touch the cup at the same time). If we assume that handovers with full cups are more challenging, then the higher number of shifts of attention during different phases of the handover could reflect the previously reported need of individuals for more action planning in more difficult tasks [33,34]. We also compare the occurrence frequency of interaction patterns across participant pairs and find one cluster of pairs that differs from others (cluster *α*). Interactions in this cluster are marked by peoples' focus and coordination of visual attention on the cup, regardless of the task difficulty. We cannot find an explanation for these clusters in terms of participant characteristics or familiarity between participants, and it would be interesting to further investigate these participant-specific differences with more data. The two largest clusters in this analysis do not reflect the two experimental treatments (empty versus full cup). This suggests that differences between interaction pairs are more substantial than the differences across treatments we found.

Interestingly, we find that participants overtly attending to their interaction partner by looking at their face were not a characteristic component of handover patterns in our data. This confirms previous work [12,13], but is in contrast with the widely held view that mutual gaze is crucial for social interactions (e.g. [37]). Note, however, that interactions in our experiment were initiated externally via a visual–auditory signal, and we therefore cannot exclude that, in a less controlled and repetitive setting, gaze is more important for coordinating handover interactions (but see [21]). Research into human–robot interactions also shows that deliberately trying to engage others in eye contact during a handover task can affect how engaged people report they are with their interaction partner [38].

While we used our methods to investigate human physical interactions, our approach is general and can be applied to social interactions in different animals. It is increasingly possible to infer behavioural states and relative positions of animals or humans from remotely recorded data [39,40]. We suggest that our approach applied to such data presents a promising way forward to investigate social interactions when a direct observation of the interactions is difficult. Furthermore, we have already argued above that investigating interaction patterns is directly relevant to research into human–robot interactions. In addition to the methodological advance our approach offers, the data we present here could be useful for the design of social robots.

An idea we have not investigated here is to identify particular interaction patterns for further study. For example, one might want to investigate the most frequent or the longest characteristic pattern found. More generally, the outcome of our analysis is a set of interaction sequences that are characteristic for one type of social interaction. Our analysis framework is general and can be applied to different types of social interactions. Assessing the similarities or differences of sequences or sets of sequences is a well-studied problem in bioinformatics [26,41]. We suggest that these techniques could be adapted to explore the similarities and differences between sets of characteristic sequences arising from different social interactions. This would present a highly novel way to quantitatively establish a taxonomy of social interactions.

## Supplementary Material

Supplementary Methods and Results from A method for detecting characteristic patterns in social interactions with an application to handover interactions

## Data accessibility

Our data and algorithms used in the analysis are deposited on the Dryad Digital Repository: http://dx.doi.org/10.5061/dryad.8j27n [42].
