# Supplementary material for: A method for detecting characteristic patterns in social interactions with an application to handover interactions
Source: R Soc Open Sci. 2017 Jan 18;4(1):160694. doi: 10.1098/rsos.160694 (PMC5319336; doi:10.1098/rsos.160694)
Supplement: Supplementary Methods and Results from A method for detecting characteristic patterns in social interactions with an application to handover interactions [file rsos160694supp1.pdf]

Supplementary information

A method for detecting characteristic patterns in social interactions with an application to handover interactions

Published in Royal Society Open Science

Authors: Nikolai W.F. Bode, Andrew Sutton, Lindsey Lacey, John G. Fennell, Ute Leonards

Supplementary methods

Experimental Methods

We conduct social interaction experiments with 25 pairs of participants. In the experiment, we ask one participants to pick up a cup from a table, hand it over to the second participant who then places it down onto the table again. Throughout the experiment, participants are allowed to talk and sit facing each other at opposite sides of the table. Two saucers are placed on the table at equal distances from the participants and approximately 0.8m apart from each other. On one of the saucers, we place a plastic cup with a metal lining on the outside. The cup is either empty, half-filled or full of dry rice, and the level of cup fullness is our experimental treatment. Participants are asked not to spill any rice. Each participant pair completes one block of trials for each treatment level (termed ‘experimental run’) and the order of treatments is determined pseudo-randomly for each participant pair to achieve counterbalancing between pairs. A computer monitor with speakers placed on one end of the table is used to signal the beginning of a handover within a block of trials by emitting a colour and tone signal. This signal indicates who out of the two participants has to pick up the cup and pass it on to the other participant, who then places the cup on the saucer at the opposite side of the table from where it was picked up. This handover interaction is repeated 100 times in each block of trials or experimental run. For each handover interaction, the participant initiating the handover (‘initiator’) is selected pseudo-randomly and the next signal is emitted approximately 1 second after the end of the previous trial. The completion of a handover is determined with the help of custom-made gloves both participants wear: a weak electric current is passed through wires on each glove; a circuit through the glove

is closed/opened when participants grasp/release the cup. Data from the closed/open circuits on the gloves are recorded, but not used in subsequent analyses.

Throughout the experiment, both participants wear a mobile eye tracker (SMI-ETG V1.8; BeGaze 3.4.46; IDF version 10; eye camera mode binocular, sample rate 30Hz; calibration area 1280x960pixels). The eye trackers are calibrated (and validated) for each participant at the start of each block of trials with a 3-point procedure and validated again at the end of the block. Data output from the eye trackers comprise a video showing the environment in front of the wearer, alongside with information on the wearer's gaze (2D coordinates for gaze fixation point on video frame, and classification of wearer's gaze into fixations, saccades and blinks based on the built-in dispersion algorithm and default values). The frame rate of the videos is 24 Hz. From these videos, we manually determine the time points (frames) when participants grasp the cup. For all videos analysed, participants' hands and the cup are visible in the eye tracker videos. We use the time interval during which both participants grasp the cup, determined for each video, to synchronise the two videos for a pair of participants in time. In addition, we manually determine for every video frame in which the gaze of a participant is fixed (fixations) whether the participant looks at the face of the other participant, at the cup, or elsewhere. Specifically, we determine that participants look at a region of interest (ROI; cup or the face of their opposite) if the gaze location is approximately within 1.5 times the radius of the ROI from the centre of the ROI (to allow for some error in the recording of the gaze fixation point). If participants look at the same ROI for two consecutive gaze fixations, we assume that they also look at this ROI for all intermediate saccades or blinks. For all saccades and blinks between two consecutive fixations on different ROIs, we assume participants look elsewhere (i.e. not on the cup or the face of their opposite). In this way we determine for each frame of the video the ROI participants' gaze is directed at.

Given the extent of manual data analysis required and substantial loss of eye tracking data for one or the other participant in a pair, we restrict analysis to the first 20 handover tasks for full and empty cups for those 11 pairs of participants with complete data sets. As the handover difficulty level in the half full condition cannot really be parametrically set between empty and full, we concentrate on the two extreme conditions only. We only use data from the first 20 handovers to keep the amount of manual analysis required manageable, but fundamentally this choice is arbitrary. We acknowledge that this selection reduces the amount of available data substantially and we cannot control for potential artefacts in the data introduced by

our selection. We therefore mainly use these data to illustrate our analysis methods. Following this procedure, we obtain data for a total of 440 handovers trials (220 each for treatment levels full and empty). For each individual handover interaction, we use data starting 0.5s before and finishing 0.5s after either participant grasps the cup. For the participant pairs included in our analysis, we present information on age, gender and familiarity of participants, as well as the order of treatment blocks in table S1. Participants do not drop the cup in any of the handover interactions included in our analysis, and only small amounts of rice are spilled when the cup is full of rice.

We check that ROI fixations are labelled consistently. For this, two independent coders separately labelled all frames for 59% of all handover interactions (260 out of 440 handover interactions). Between the data used in our analysis and these separately coded data, we find 95% agreement in ROI fixation labelling across frames.

Supplementary results

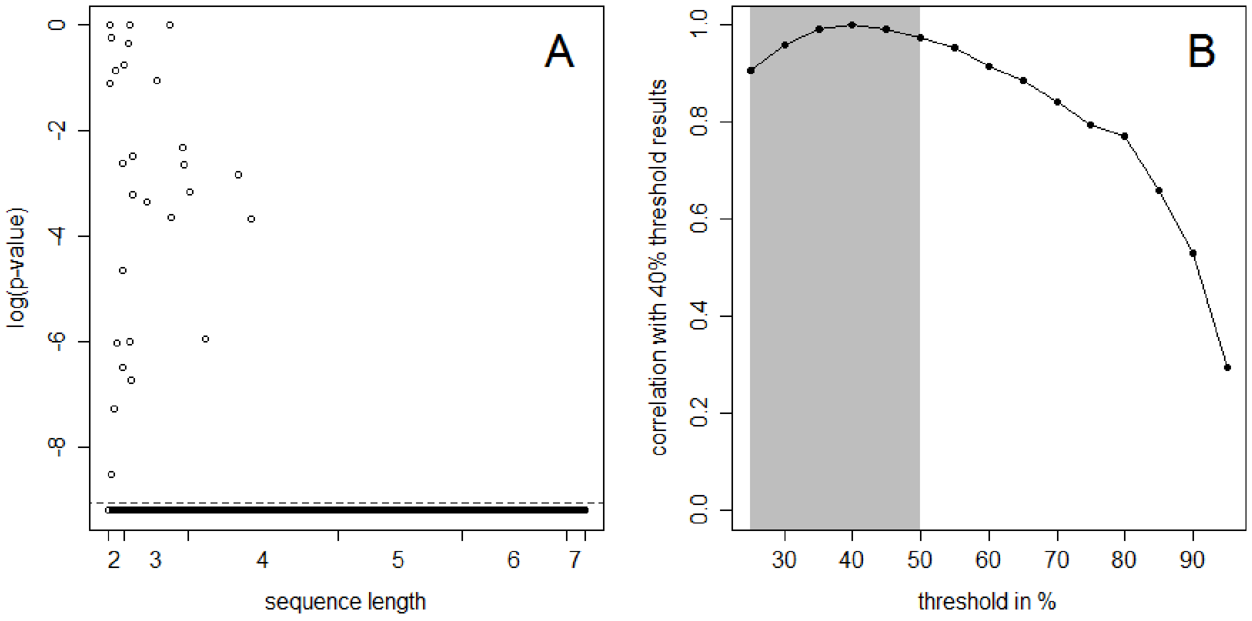

Figure S1: (A) All but 28 of characteristic interaction sequences (CIS) used in our analysis occur more frequently in the observed behavioural state sequences than expected if individual-level behavioural states are re-arranged randomly. The p-values arising from a permutation test are shown for all CISs included in the reference set (10,000 permutations, see main text section 3.4 for details). CISs are ordered according to length, but individual patterns are not shown for clarity of illustration. The dashed line shows the significance threshold of 0.1 divided by the number of patterns (m=852), using a Bonferroni correction to account for multiple comparisons. (B) Spearman-rank correlation between upper-triangular parts of Euclidean distance matrices between CIS occurrence count vectors for a range of thresholds used for finding the CISs (see section 4.1 in the main text). High correlations indicate that differences between occurrence counts are similar for different threshold values. We find high correlations for a broad range of thresholds, suggesting our findings are robust to the threshold value chosen.

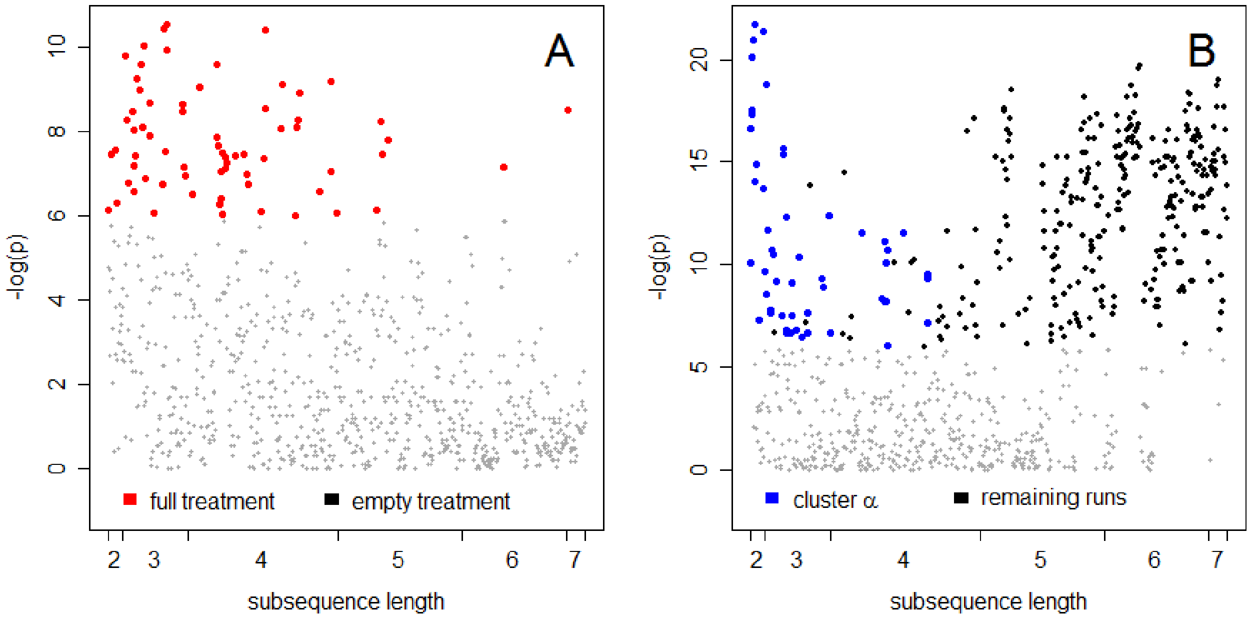

Figure S2: P-values for whether a characteristic interaction sequence (CIS) occurs more frequently in a treatment or cluster. P-values for all CISs are shown. CISs are ordered according to length, but individual patterns are not shown for clarity of illustration. For each CIS, we construct a contingency table for how often it occurs in the behavioural state sequences (BSSs) for different treatments or clusters. We use chi-squared tests on these tables to determine p-values and use a significance threshold of *0.001/m* (where *m=852*, the number of CISs in the reference set; see section 4.1 in the main text). For each CIS, we determine if it occurs more frequently in the treatment level or cluster under consideration. In this way, we obtain a set of CISs that are significantly more likely to occur for a particular treatment level (panel A) or in a particular cluster (panel B). We find no CISs that occur significantly more frequently for the empty treatment, but 67 for the full treatment (panel A). We find 51 CIS that occur more frequently in cluster *α* (blue in panel B). The remaining CIS with significant p-values in panel B (in black) are ones which are under-represented in experimental runs in cluster *α*. We show the negative of the logarithm with base 10 of p-values. All p-values larger than the significance threshold are shown in grey.

Pair	01	02	03	04	05	06	07	08	09	10	11
**Age (years)**	18, 19	20, 20	18, 19	19, 19	19, 31	18, 18	19, 19	18, 18	18, 17	18, 18	29, 19
**Gender (f/m)**	m, m	f, f	f, m	f, f	f, f	m, m	f, f	f, f	f, f	f, f	f, f
**Familiarity**	0.01	0.92	0.0	0.0	0.0	0.75	0.64	0.0	0.0	0.83	0.0
**Treatment Order**	E, F	E, F	F, E	E, F	F, E	E, F	E, F	F, E	E, F	F, E	E, F

Table S1: Age and gender for each participant pair included in our analysis. The fourth row shows a familiarity score for the participant pair (1 implies participants are very familiar, 0 implies they have never met before). This score was computed by taking the average of the familiarity the two participants reported via a Visual Analogue Scale. The fifth row shows the order in which trials with empty cup (‘E’) and full cup (‘F’) treatments were conducted for each participant pair. Numbers refer to the same participant pairs shown in figure 2 in the main text. All participants were right-handed, apart from one participant in pair 9 who was left-handed.

Feature	Fraction of CISs that contain feature
Contains 1	0.99
Contains 2	0.00
Contains 3	0.57
Contains 4	0.99
Contains (1,1)	0.87
Contains (2,2)	0.00
Contains (3,3)	0.01
Contains (4,4)	0.40
Contains change in grasp, e.g. (1,4)(4,1)	0.41
Contains change in visual attention on cup, e.g. (1,3)(3,1)	0.04
Average pattern length	4.6

Table S2: Features of the characteristic interaction sequences (CISs) found in our data. We show the fraction out of all 852 CISs found that contain particular features, as well as the average CIS length across all CISs. We show the following features: behavioural state occurrence, synchronous occurrences of behavioural states, change in grasp (i.e. first one and then the other participant holds the cup), change in visual attention on the cup (i.e. first one and then the other participant looks at the cup) and average pattern length. For example, no CIS contained the behavioural state 2 (i.e. look at other person’s face). A file containing all CISs is included as supplementary information. The behavioural states are as follows: 1 - participant looks elsewhere; 2 - participant looks at the face of the other participant; 3 - participant looks at the cup; 4 - participant grasps the cup.
